# Pharmacokinetic Characterization and Tissue Distribution of Fusion Protein Therapeutics by Orthogonal Bioanalytical Assays and Minimal PBPK Modeling

**DOI:** 10.3390/molecules25030535

**Published:** 2020-01-26

**Authors:** Hiroshi Sugimoto, Susan Chen, Mark G. Qian

**Affiliations:** Takeda Pharmaceuticals International Co., Drug Metabolism and Pharmacokinetics, 35 Landsdowne Street, Cambridge, MA 02139, USA; susan.chen@takeda.com (S.C.); mark.qian@takeda.com (M.G.Q.)

**Keywords:** fusion protein, immunocapture-LC/MS, LBA, minimal PBPK model

## Abstract

Characterization of pharmacokinetic (PK) properties and target tissue distribution of therapeutic fusion proteins (TFPs) are critical in supporting in vivo efficacy. We evaluated the pharmacokinetic profile of an investigational TFP consisting of human immunoglobulin G4 fused to the modified interferon alpha by orthogonal bioanalytical assays and applied minimal physiologically based pharmacokinetic (PBPK) modeling to characterize the TFP pharmacokinetics in mouse. The conventional ligand binding assay (LBA), immunocapture-liquid chromatography/tandem mass spectrometry (IC-LC/MS) detecting the human IgG4 peptide or the interferon alpha peptide were developed to measure the TFP concentrations in mouse plasma and tumor. The minimal PBPK model incorporated a tumor compartment model was used for data fitting. The plasma clearance measured by LBA and IC-LC/MS was comparable in the range of 0.5–0.6 mL/h/kg. However, the tumor exposure measured by the generic human IgG4 IC-LC/MS was significantly underestimated compared with the interferon alpha specific IC-LC/MS and LBA. Furthermore, the minimal PBPK model simultaneously captured the relationship between plasma and tissue exposure. We proposed the streamlined practical strategy to characterize the plasma exposure and tumor distribution of a TFP by both LBA and IC-LC/MS. The minimal PBPK modeling was established for better understanding of pharmacokinetic profile of investigational TFPs in the biotherapeutic discovery.

## 1. Introduction

The advancement of genetic translation and recombinant technologies have enabled to develop fusion proteins targeting multiple targets to achieve better efficacy, safety, and pharmacokinetic profile such as extended half-life, targeted distribution, and enhanced pharmacologic activity. A common approach of therapeutic proteins is to fuse a human IgG fragment crystallizable (Fc) region, which is responsible for the neonatal Fc receptor (FcRn) binding by antibody recycling for a longer half-life [1]. However, the pharmacokinetic property of TFP is less well characterized than the conventional monoclonal antibody-based biotherapeutics. 

The primary determinants of TFPs disposition are renal elimination, target mediated drug disposition, FcRn mediated recycling, anti-drug antibody clearance, catabolism and tissue distribution followed by catabolism within tissue. The exposure at the site of action, i.e., in the tumor tissue where the therapeutic target antigen expresses is one of the significant interests for evaluating anti-cancer biotherapeutics [2]. However, the quantification of TFPs in tissues has unique challenges such as efficient extraction from tissues and the requirement of high sensitivity for detection since tissue concentrations of TFPs are usually much lower than plasma concentrations [3,4]. In general, the tissue-to-blood concentration ratio ranges from 0.04 to 0.16 for monoclonal antibody based biotherapeutics because of the large molecular weight of the protein which limits its distribution from vascular to interstitial spaces of tissues [5,6].

The predominant bioanalytical platform for monitoring the concentration of therapeutic proteins is LBA-based assay due to its high sensitivity, selectivity and throughput. In addition, the assay can potentially provide the information regarding the molecular integrity and functionality of therapeutic proteins. For example, LBA-based assay captures the concentration of an intact TFP with anti-Fc capture antibody and anti-therapeutic protein detection antibody. However, LBA-based tissue analysis may be challenging due to nonspecific binding and endogenous interference that negatively impact the assay robustness [7,8]. Recently, the IC-LC/MS technology has been increasingly accepted as a reliable assay platform with unique advantages of multiplexing capabilities, less stringency on reagent requirements, and minimization of potential endogenous interference. In addition, signature peptide detection for selected protein motifs can deconvolute concentrations of target analytes in multiple varied co-existent forms [9]. The intact assay should capture active TFP molecule whose functional domain is not truncated or blocked by anti-drug antibody. The IC-LC/MS-based assay with anti-Fc capture antibody and the specific signature peptide detection in the therapeutic protein will also provide the concentration of an intact TFP. On the other hand, the generic capture reagents such as anti-Fc, protein A/G and L combined with human IgG signature peptide detection also allows the generic human IgG quantification especially at the early drug discovery stage. IC-LC/MS and LBA-based assays have been evolved as orthogonal analytical tools for monitoring pharmacokinetic profiles of biotherapeutics in systemic circulation [10]. In this report, we aimed to establish both generic/specific IC-LC/MS and LBA-based assays to elucidate the pharmacokinetic profile in plasma and tissue of the TFP comprising of human IgG4 targeting cell surface CD38 fused to a modified form of human interferon alpha.

To facilitate the understanding of the mechanism of TFP tissue distribution, the physiologically-based pharmacokinetic (PBPK) model is a useful tool which could potentially provide mechanistic characterization of drug disposition by taking physiological parameters and anatomical and physical descriptions into account. Alternatively, minimal PBPK models inherit the essence from the full PBPK with simplified differential equations, but still provides physiologically relevant parameters [11]. In particular, recent established PBPK models captured the fundamental distribution mechanism of monoclonal antibody-based biotherapeutics such as lymphatic convection, drainage, and interstitial fluid as the primary extravascular distribution space [12,13]. In this study, we have modified the original minimal PBPK model with an addition of a tumor compartment to delineate the relationship of plasma and tumor pharmacokinetics of TFPs.

## 2. Results and Discussion

### 2.1. Strategy to Characterize the Pharmacokinetic Property of TFPs by LBA and IC-LC/MS

**Figure 1:**
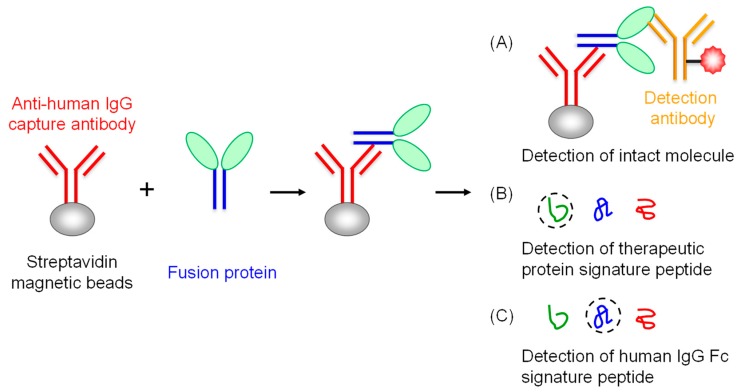
Ligand binding assay (LBA) and IC-LC/MS-based assays for the TFP. The LBA assay measures the concentration of the intact TFP with an anti-human IgG (h+l) for capture and the labeled anti-therapeutic protein antibody for detection (**A**). The IC-LC/MS assay determines the concentration of the intact TFP based on the signature peptide from the therapeutic protein (**B**) or human IgG Fc (**C**).

The LBA assay was developed to measure the intact TFP molecule with an anti-Fc antibody for capture antibody and a ruthenylated mouse anti-interferon alpha antibody for detection antibody (Figure 1A). Although a generic human Fc assay may be used for the intact TFP, the assay cannot differentiate the intact molecule and any circulating variant forms, e.g., catabolites chipped off the therapeutic protein domain in the molecule. In addition, we developed an IC-LC/MS assay to measure TFP by signature peptides specific to the therapeutic protein (Figure 1B) and generic human IgG Fc (Figure 1C). The LC/MS based assay served as an orthogonal tool for the LBA assay since the LBA assay may be subject to the interference from the nonspecific binding and the endogenous free circulating antigen.

### 2.2. Selection of Signature Peptides to Quantify the Human IgG4 and Interferon Alpha

The proteolytic signature peptides for human IgG4 (SLSLSLGK) and interferon alpha (EDSILAVR) were selected according to the selection criteria to enhance the selectivity and minimize the potential interference and post-translational modification [14,15]. Briefly, the signature peptide needs to be unique in the sequence. The peptide length should be typically 7–15 amino acids. The reactive residues such as Cys, Met, *N*-terminal Gln, Asn Trp should be avoided. At last, residues with the potential posttranslational modifications [e.g., phosphorylation, *N*-glycosylation (NXS/T)] should be avoided. The doubly charged precursor ions with y6 fragment ion for SLSLSLGK and y4 fragment ion for EDSILAVR were selected for quantification of TFP. Although the universal surrogate peptide of VVSVLTVLHQDWLNGK in human IgG1 and IgG4 Fc region has been reported for use in cynomolgus monkey PK study [16], the peptide was not applicable to our TFP because QC samples did not meet the acceptance criteria.

### 2.3. Workflow of Tumor Sample Preparation and Immunocapture for LBA and IC-LC/MS

**Figure 2:**
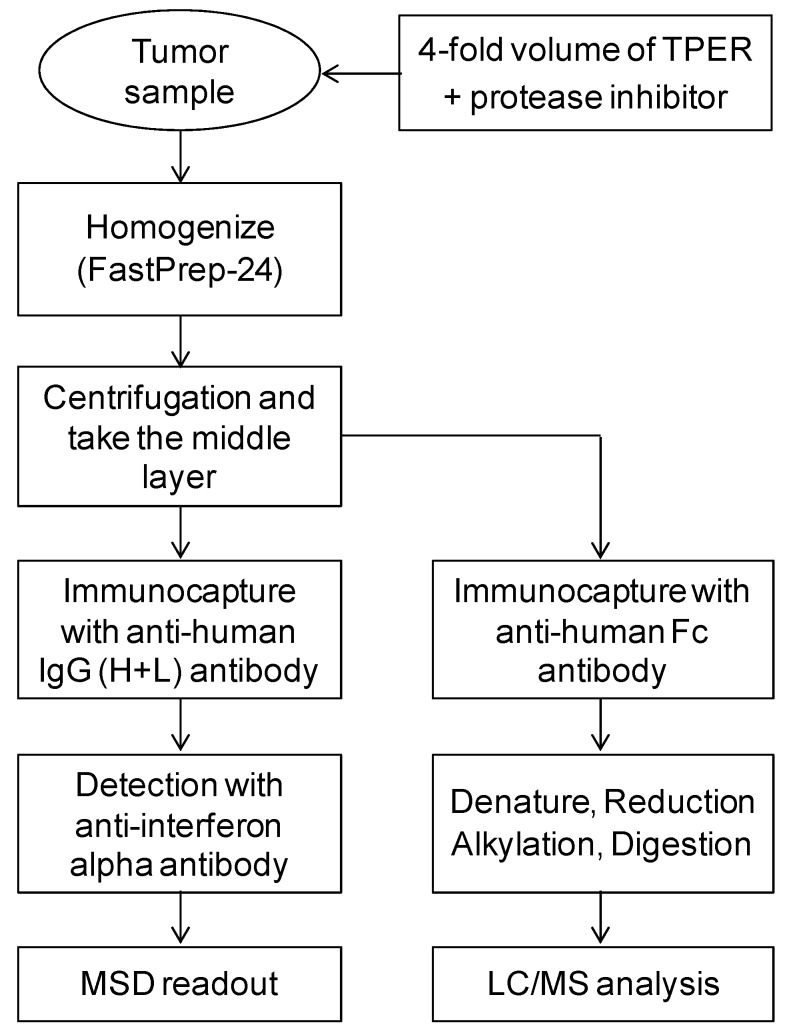
Workflow of tumor sample preparation and immunocapture for LBA and IC-LC/MS.

A major challenge for quantitative tumor analysis of TFP molecule is the matrix effect which may compromise assay sensitivity and selectivity. To overcome the challenge, the tissue sample preparation and further immunocapture process followed by LBA and LC/MS were optimized. The streamlined procedure was summarized in Figure 2. Tumor tissue samples were homogenized in 4-fold volume of the tissue protein extraction reagent containing 1% of protease inhibitor cocktail with FastPrep-24 homogenizer. It is important to avoid taking lipid surface layer after the centrifuge and collecting the middle portion of supernatant for the further immunocapture process for LBA or IC-LC/MS. Immunocapture purification is important in order to get rid of interfering matrix components and protease inhibitors which may inhibit the trypsinization efficiency of TFP. TFP concentrations were determined by both LBA- and IC-LC/MS-based generic human IgG4 peptide (generic IC-LC/MS) or specific interferon alpha (specific IC-LC/MS) peptide assays. The assay precision (%CV) and accuracy (%RE) for LBA and each IC-LC/MS in plasma and tumor sample met the acceptance criteria (Table 1).

### 2.4. The Plasma and Tumor Concentration Versus Time Profile in Mice after a Single i.v. Administration of the TFP

**Figure 3:**
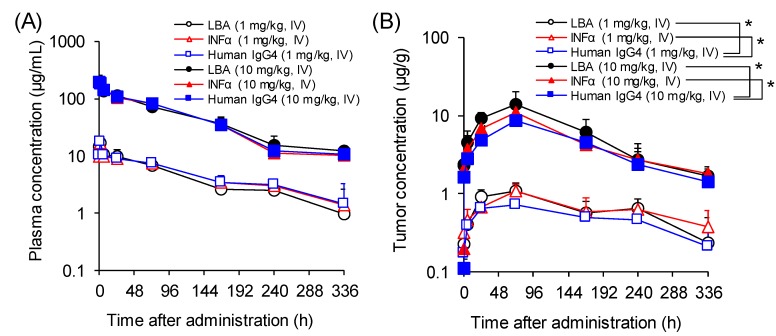
Plasma (**A**) and tumor (**B**) concentration versus time profile in mice after a single i.v. administration of TFP. The plasma and tumor concentration versus time profile in mice, after a single i.v. administration of the TFP at doses of 1 and 10 mg/kg, determined by LBA (circle), IC-LC/MS assay using interferon alpha specific peptide (triangle) and human IgG4 generic peptide (square). The values were expressed as mean + S.D. (*n* = 3). Data were analyzed by one-way ANOVA followed by Dunnett’s post hoc test. Statistically significance* was set at *p* < 0.05 for all tests.

TFP was intravenously administered to mice at the doses of 1 and 10 mg/kg. The plasma and tumor concentrations were determined by LBA, generic and specific IC-LC/MS. The pharmacokinetic profiles and parameters of the TFP were summarized in Figure 3 and Table 2, respectively. The plasma AUC values calculated from TFP concentrations determined by LBA assay were similar to those determined by the IC-LC/MS assay without any statistical difference. The plasma clearance of the TFP was in the range of 0.5–0.6 mL/h/kg at doses of 1 and 10 mg/kg suggesting that the TFP molecule is relatively stable in mouse plasma. Since the clearance and half-life of naked interferon alpha-2b in human were reported as 231.2 mL/h/kg and 2–4 h, respectively [17], the introduction of the Fc binding moiety to interferon has successfully extended the half-life of the TFP. In addition, the TFP with an Fc binding moiety whose molecular weight of >150 kDa is less likely subject to renal elimination due to the renal glomerular barrier [18]. On the other hand, although tumor AUC values calculated by LBA and specific IC-LC/MS were similar, tumor AUC values determined by the generic IC-LC/MS assay was statistically significantly lower (approximately 33%) than by the specific assays (Figure 3B). A plausible explanation may be due to the matrix effect on the trypsinization efficiency of the TFP in the tumor homogenate. In addition, the variation in the extent of C-terminal lysine residue which has been reported to lead the antibody production lots with different charge distribution may have been contributed this phenomena [19,20]. In terms of the TFP exposure in tumor tissue where the target antigen is more highly expressed than those in other tissues, higher concentrations may be observed due to the saturable binding with the TFP and target antigen on the plasma membrane of tumor cells. The measured tumor-to-plasma concentration ratio ranged from 0.136 to 0.156 which is comparable with the value reported in the characteristically low target expressing tumors or when the dose is above the target saturation level [4].

### 2.5. Minimal PBPK Modeling to Describe the Relationship of Plasma and Tumor Pharmacokinetics of the TFP

**Figure 4:**
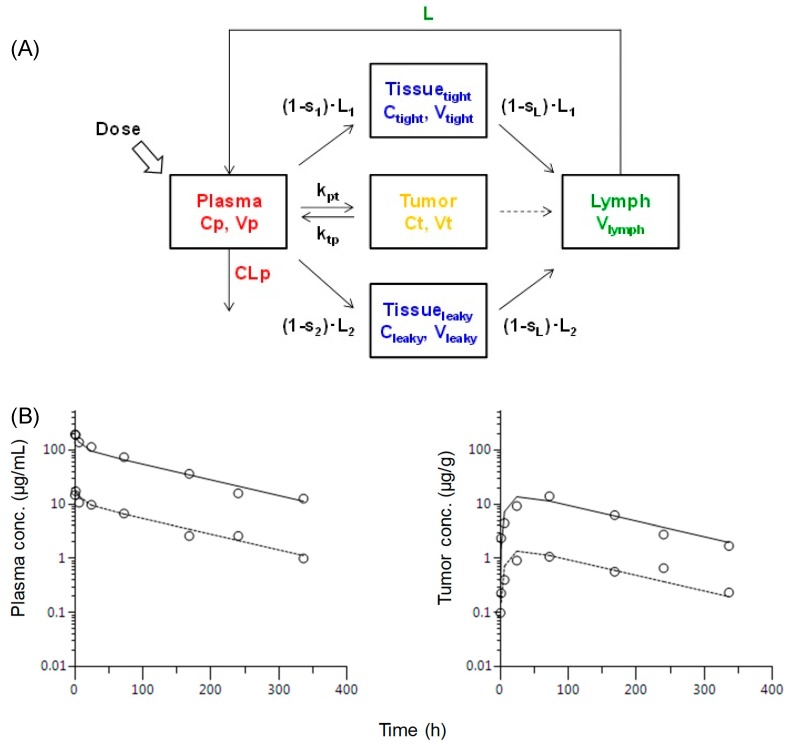
Minimal physiologically based pharmacokinetic (PBPK) model to describe the TFP molecule exposure in plasma and tumor: (**A**) The minimal PBPK model to describe the TFP exposure. (**B**) The experimental data (mean value, open circle) and minimal PBPK model based fitting pharmacokinetic profile in plasma and tumor in mice after a single i.v. administration of TFP at doses of 1 (dotted line) and 10 (solid line) mg/kg.

A minimal PBPK model consists of the physiologically relevant parameters with reduced model complexity while maintaining the mechanistic understandings for compartments of interest. The recently proposed model considers the fundamental monoclonal antibody distribution mechanism such as diffusion and lymphatic convection as the primary pathway to mediate the transcapillary escape rate [12]. The extravascular distribution is considered to be primarily determined by the interstitial fluid [12,21]. The relationship of plasma and tumor exposure of TFP measured by the LBA assay in this study was further investigated by the minimal PBPK model. In this model, the reflection coefficients, elimination rate constant of k_pt_ (plasma to tumor) and k_tp_ (tumor to plasma) need to be fitted in the minimal PBPK model. To describe the relationship of plasma and tumor pharmacokinetics of the TFP, the tumor compartment was incorporated in the modified minimal PBPK model because the apparent linear pharmacokinetic profile in plasma and tumor (Figure 3) suggested that the nonlinear pharmacokinetic model such as a saturable target-mediated drug disposition (TMDD) model [22] is not a suitable model in this case. In addition, neither the association, dissociation rate constants (k_on_, k_off_) of drug-target complexes, the target biosynthesis nor degradation rates (k_syn_, k_deg_) were defined to incorporate into the typical TMDD model [23]. The modified PBPK model simultaneously captured the experimental data in plasma and tumor in mice after a single i.v. administration of the TFP at the doses of 1 and 10 mg/kg (Figure 4). The pharmacokinetic parameters used in the minimal PBPK model are summarized in Table 3. Previously, multiple elimination pathways have been reported for monoclonal antibody based biotherapeutics such as non-specific pinocytosis, catabolism, saturable TMDD and anti-drug antibody (ADA)-mediated clearance [24]. Although the minimal PBPK model without the clearance pathway in tight, leaky tissue and tumor compartment captured the experimental data well, the incorporation of target-mediated drug disposition model may be the next step when the microscopic parameters to describe the target binding and turnover kinetics become available. 

## 3. Materials and Methods

### 3.1. Chemicals and Reagents

The proprietary TFP, consisting of human immunoglobulin G4 (human IgG4) targeting the cell surface CD38 fused to a modified form of human interferon alpha, was prepared in-house. Anti-CD38 and human interferon alpha part were cloned into the pTT5 mammalian expressing vector [26]. Anti-human IgG (Fc specific) highly cross-adsorbed biotinylated antibody, DL-dithiothreitol (DTT), formic acid, iodoacetamide, and triethylammonium bicarbonate buffer were purchased from Sigma Aldrich (St. Louis, MO, USA). Sequencing grade modified trypsin was from Promega (Fitchburg, WI, USA). The signature peptides for human IgG4 (SLSLSLGK and SLSLSLG[K]) and interferon alpha (EDSILAVR and EDSILAV[R]), where [R] and [K] indicate ^13^C_6_^15^N_4_-R and ^13^C_6_^15^N_2_-K, respectively), Thermo KingFisher™ magnetic beads processor, T-PER tissue protein extraction reagent, and Halt protease inhibitor cocktail were from Thermo Fisher Scientific (Waltham, MA, USA). Dynabeads™ M-280 Streptavidin was from Invitrogen (Carlsbad, CA, USA). AffiniPure goat anti-human IgG (H+L) antibody is from Jackson ImmunoResearch (West Grove, PA, USA). MSD GOLD SULFO-TAG NHS-Ester is from Meso Scale Discovery (Rockville, MD, USA). Mouse anti-interferon alpha 2b antibody is from Abcam (Cambridge, MA, USA). FastPrep-24 is from MP Biomedicals (Santa Ana, CA, USA). 

### 3.2. In Vivo Human Derived Xenograft Tumor Studies in Mice

All animal research and veterinary care were performed in accordance with the Guide for the Care and Use of Laboratory Animals under approved protocols of the Takeda Boston Institutional Animal Care and Use Committee in a facility accredited by the Association for Assessment and Accreditation of Laboratory Animal Care International (AAALAC). The IACUC protocol number is 17-05-198. Healthy female immuno-deficient (SCID) mice were housed in a temperature- and humidity-controlled room with a 12 hour light/dark cycle with a standard diet and water ad libitum. For xenograft experiments, the mice were subcutaneously inoculated in flank with human myeloma cell line LP-1 cells (passage No. 8) at the number of 5.0 × 10^6^ cells/mouse. Mice were received a single intravenous administration of the TFP at the doses of 1 and 10 mg/kg when the tumor volume reached at 300–800 mm^3^. Mice were sacrificed at designated time points (5 min, 1, 6, 24, 72, 168, 240, and 336 h, three mice at each time point) after the administration and plasma samples were collected and tumor samples were resected and weighted. Samples were snap frozen and stored under −80 °C until sample analysis.

### 3.3. Instrumentation and Experimental Conditions for Ligand Binding Assay

The ligand binding assay (LBA) was conducted using MESO QuickPlex SQ 120 (Meso Scale Diagnostics, Rockville, MD, USA). Goat anti-human IgG (H+L) polyclonal antibody was used as the capture antibody and TFP as the reference standard. The standard samples, quality controls and study samples in mouse plasma are diluted in 3%BSA in PBS with 0.05% Tween 20 to achieve 4000-fold minimum required dilution. The captured TFP was detected using sulfo-tagged anti-interferon alpha 2b. The electroluminescence signal was measured by an MSD plate reader. The calibration standards ranged from 0.586–300 µg/mL for plasma and 0.039–10 µg/g for tumor, respectively. The precision and accuracy (three to four replicates) were evaluated with the TFP spiked into control biomatrix at levels of 3 (low quality control, LQC), 30 (middle quality control, MQC), and 240 (high quality control, HQC) μg/mL for plasma and 0.3 (LQC), 2 (MQC), and 8 (HQC) μg/g for tumor. The results were analyzed using a four-parameter logistic (4PL) algorithm with 1/y weighted regression. 

### 3.4. Sample Preparation Procedure for LC/MS

The stock solutions of TFP were serially diluted with blank mouse plasma and CD38 negative control tumor homogenate to prepare calibration curves ranged from 0.10–100 µg/mL for plasma and range from 0.25–50 µg/g for tumor, respectively. The precision and accuracy (five replicates) were evaluated with the TFP spiked into control biomatrix at the levels of 0.3 (LQC), 2 (MQC), and 80 (HQC) μg/mL for plasma and 0.3 (LQC), 2 (MQC) μg/g for tumor. The plasma sample with higher than upper limit of quantification limit was diluted in blank mouse plasma. The sample pretreatment procedure was described previously with slight modifications [27]. Briefly, 100 µL of PBS was added into a 96-well plate followed by 100 µL/per well of biotinylated anti-human Fc monoclonal antibody beads (0.5 mg anti-human Fc beads) and 8 µL of plasma and 25 µL of tumor homogenate samples. The samples were incubated at room temperature for 45 min with gentle mixing. The plate was processed with KingFisher™ Flex Magnetic Particle Processor to transfer beads for binding and washing with PBST, PBS and 10% acetonitrile, respectively. The analyte was eluted with acetonitrile/30 mmol/L HCl (1:3, *v*/*v*) and adjust pH to 8. After the reduction and alkylization, trypsin was added to the plate and incubated at 37 °C for overnight. The protein digestion was stopped by acidification with formic acid. After adding the internal standard and acetonitrile, and centrifugation for 10 min at 3000 rpm, the supernatant was transferred to and injection plate and blown dry under a nitrogen steam at 40 °C. The samples were reconstituted with 5% acetonitrile/0.1% formic and subjected to LC/MS analysis.

### 3.5. Method qualification

LBA and IC-LC/MS assay performance was evaluated based on fit-for-purpose approach with limited precision, accuracy and specificity as discussed previously [28]. Briefly, the LBA-based assay standard curve was fit using a 4-parameter logistic regression (4PL) algorithm with 1/y weighted regression using SoftMax Pro 7 Software (Molecular Devices, San Jose, CA). For IC-LC/MS-based assay, a linear model fitted by least-squares linear regression with weighting factor 1/x^2^ was used to describe the calibration curve based on the area ratios of analyte to internal standard versus the nominal concentrations of analyte. The acceptance criteria of the relative error (%RE) of the back-calculated concentrations to the nominal concentrations was set as within ±20% of nominal values (for lower limit of quantification, LLOQ; ±25%). The acceptance criteria of the coefficient of variation (%CV) was set within ±20%. The intra-day precision and accuracy were assessed in pooled QC samples. 

### 3.6. Instrumentation and Conditions for LC/MS Analysis

The LC/MS consisted of an ultra-high-performance liquid chromatograph system (Shimadzu, Kyoto, Japan) and a hybrid triple quadrupole linear ion trap mass spectrometer QTRAP^®^ 5500 system (Sciex, Framingham, MA, USA). Samples were loaded on Aeris™ PEPTIDE XB-C18 100 Å LC Column (1.7 µm, 50 x 2.1 mm, Phenomenex, Torrance, CA, USA) set at 50 °C. Gradient elution was conducted using 0.1% formic acid (solvent A) and acetonitrile containing 0.1% formic acid (solvent B). The stepwise gradient program was used as follows; 0–0.4 min, B 5%; 0.4–3.2 min, B 5–50%; 3.2–3.3 min, B 50–90%; 3.3–4.1 min, B 90%; 4.1–4.2 min, B 5%; 4.2–5 min, B 5%. The flow rate of the mobile phase was 0.5 mL/min. Multiple reaction monitoring (MRM) was performed with unit resolution for Q1 and Q3. The ionization mode was electrospray ionization (ESI) in positive ion mode and the source temperature was set at 600 °C. A nitrogen curtain gas, ion source gas 1, ion source gas 2 and ion source voltage were set at 25 psi, 50 psi, 50 psi and 5500 V, respectively. The optimized mass transition and condition are summarized in Table 4. Mass spectrometric data were acquired and processed using the software Analyst version 1.7 (Sciex). The back-calculated concentrations were described in three significant figures. The IS normalized peak areas of the surrogate peptides were used to describe the calibration curve fitted by least-squares linear regression with weighting factor 1/x^2^. The criteria of the relative error (%RE) of the back-calculated concentrations to the nominal concentrations were set as within ±20% of nominal. 

### 3.7. Pharmacokinetic Analysis

The minimal PBPK model is based on the previously published model [12] with an addition of tumor compartment to capture the relationship between plasma and whole tumor concentrations of TFP in this study. The detailed differential equations are as follows:

$$\frac{{\mathrm{dX}}_{p}}{\mathrm{dt}}=-{\mathrm{CL}}_{p}\cdot C_{p}-L_{1}\cdot \left(1-S_{1}\right)\cdot C_{p}-L_{2}\cdot \left(1-S_{2}\right)\cdot C_{p}+C_{\mathrm{Lymph}}\cdot L-k_{\mathrm{pt}}\cdot X_{p}+k_{\mathrm{tp}}\cdot X_{t}$$

$$\frac{{\mathrm{dX}}_{p}}{\mathrm{dt}}=-{\mathrm{CL}}_{p}\cdot C_{p}-L_{1}\cdot \left(1-S_{1}\right)\cdot C_{p}-L_{2}\cdot \left(1-S_{2}\right)\cdot C_{p}+C_{\mathrm{Lymph}}\cdot L-k_{\mathrm{pt}}\cdot X_{p}+k_{\mathrm{tp}}\cdot X_{t}$$

$$\frac{{\mathrm{dX}}_{\mathrm{tight}}}{\mathrm{dt}}=L_{1}\cdot \left(1-S_{1}\right)\cdot C_{p}-C_{\mathrm{tight}}\cdot L_{1}\cdot \left(1-S_{L}\right)$$

$$\frac{{\mathrm{dX}}_{\mathrm{leaky}}}{\mathrm{dt}}=L_{2}\cdot \left(1-S_{2}\right)\cdot C_{p}-C_{\mathrm{leaky}}\cdot L_{2}\cdot \left(1-S_{L}\right)$$

$$\frac{{\mathrm{dX}}_{\mathrm{lymph}}}{\mathrm{dt}}=C_{\mathrm{tight}}\cdot L_{1}\cdot \left(1-S_{L}\right)+C_{\mathrm{leaky}}\cdot L_{2}\cdot \left(1-S_{L}\right)-C_{\mathrm{lymph}}\cdot L$$

$$\frac{{\mathrm{dX}}_{t}}{\mathrm{dt}}=k_{\mathrm{pt}}\cdot X_{p}-k_{\mathrm{tp}}\cdot X_{t}$$

$$C_{p}=\frac{X_{p}}{V_{p}},{\;C}_{\mathrm{tight}}=\frac{X_{\mathrm{tight}}}{V_{\mathrm{tight}}},{\;C}_{\mathrm{leaky}}=\frac{X_{\mathrm{leaky}}}{V_{\mathrm{leaky}}},{\;C}_{\mathrm{lymph}}=\frac{X_{\mathrm{lymph}}}{V_{\mathrm{lymph}}},{\;C}_{t}=\frac{X_{t}}{V_{t}}$$

$$V_{\mathrm{tight}}=0.65\times \mathrm{ISF}\times K_{p},{\;V}_{\mathrm{leaky}}=0.35\times \mathrm{ISF}\times K_{p}$$

$${\;L}_{1}=0.33\times L,{\;L}_{2}=0.67\times L$$

where C_p (plasma)_, C_tight_, C_leaky_, C_lymph_ and C_t (tumor)_ indicate substrate concentration in each compartment. V_p (plasma)_, V_tight_, V_leaky_, V_lymph_ and V_t (tumor)_ indicate plasma, interstitial fluid (ISF) and tumor tissue volume. k_pt (plasma to tumor)_ and k_tp (tumor to plasma)_ are elimination rate constants from plasma to tumor and tumor to plasma, respectively. L is total lymph flow with a combination of L_1_ and L_2_ for tight and leaky tissue, respectively. The recycled TFP in the tumor compartment back into the lymph node compartment is considered to be negligible [23]. All initial conditions are set as 0 except for C_p_. The pharmacokinetic analysis and minimal PBPK modeling development were performed using Phoenix™ WinNonlin^®^, version 7.0 (Pharsight Corp, Mountain View, CA, USA). The AUC were calculated by non-compartmental analysis (NCA) using the linear trapezoidal rule. Clearance was calculated by Dose / AUC. The T_1/2_ was calculated from the actual values by the least-squares method.

### 3.8. Statistics

The statistical significance of the difference between mean values was tested using a one-way analysis of variance followed by Dunnett’s comparison test. Differences with a *p*-value less than 0.05 were considered statistically significant.

## 4. Conclusions

Therapeutic fusion proteins (TFPs) are emerging biotherapeutic modalities with an advantage of half-life extension through introduction of an Fc binding moiety, yet many biologically active proteins have short half-life because of fast renal excretion. In this study, we proposed the bioanalytical strategy to characterize the plasma exposure and tumor distribution of TFPs. Since an IC-LC/MS assay with a generic human IgG4 signature peptide significantly underestimated the tumor distribution of TFP, we proposed the strategy to characterize the pharmacokinetic properties of TFPs in the nonclinical in vivo study by specific LBA or IC-LC/MS to detect the whole TFP molecule including interferon alpha rather than the human IgG4 backbone of TFP. In addition, the relationship between measured plasma and tumor pharmacokinetics was well delineated by the modified minimal PBPK model with incorporation of a tumor compartment. These described assays and modeling work may be useful in general for better understanding of pharmacokinetic profile of investigational TFPs in the biotherapeutic discovery.

## Figures and Tables

**Table 1:** Assay precision and accuracy for LBA and human IgG4 / interferon alpha signature peptide by IC-LC/MS in mouse plasma and tumor.

Assay Platform	Signature Peptide	Matrix		Nominal Conc. (μg/mL or g)	Intra-Assay		
					Average Conc. (μg/mL)	CV (%)	RE (%)
LBA	-	Plasma	LQC	3.00	2.79	8.1	−7.0
			MQC	30.0	32.3	12.1	7.6
			HQC	240	278	12.9	15.6
		Tumor	LQC	0.300	0.301	8.0	0.3
			MQC	2.00	2.14	5.7	7.0
			HQC	8.00	8.27	4.1	3.3
IC-LC/MS	Human IgG4	Plasma	LQC	0.300	0.353	4.1	17.7
			MQC	2.00	2.20	7.6	10.0
			HQC	80.0	85.2	2.7	6.5
		Tumor	LQC	0.300	0.316	8.2	5.2
			MQC	2.00	1.98	2.3	−0.8
	Interferon alpha	Plasma	LQC	0.300	0.334	10.2	11.2
			MQC	2.00	2.18	5.4	9.1
			HQC	80.0	88.1	4.1	10.1
		Tumor	LQC	0.300	0.329	15.4	9.5
			MQC	2.00	1.67	6.7	−16.6

**Table 2:** The comparison of pharmacokinetic parameter based on LBA and IC-LC/MS-based assay.

		AUC (h*μg/mL)			CL (mL/h/kg)			Cmax (μg/mL)			T _1/2_ (h)
			IC-LC/MS			IC-LC/MS			IC-LC/MS		
	Dosing	LBA	INFα	IgG4	LBA	INFα	IgG4	LBA	INFα	IgG4	
Plasma	IV 1 mg/kg	1470	1600	1650	0.624	0.539	0.527	17.3	17.3	17.6	98.1
	IV 10 mg/kg	16200	16000	16400	0.559	0.593	0.568	194	202	194	90.8
Tumor	IV 1 mg/kg	229	227	168				2.31	1.93	1.57	
	IV 10 mg/kg	2210	1730	1480				14.0	10.9	8.67	

Comparison of pharmacokinetic parameter based on LBA and IC-LC/MS-based assay. TFP concentrations in mouse plasma and LP-1 tumor were measured by electrochemiluminescence LBA assay capturing with anti-human IgG (h+l) and detecting with ruthenylated anti-human interferon alpha or immunocapture-LC/MS assay capturing with anti-human IgG (Fc specific) and detecting with interferon alpha and human IgG4 specific peptides. The values were expressed as mean values because the plasma and tumor samples were harvested as terminal sampling from each animal.

**Table 3:** The pharmacokinetic parameters used in the minimal PBPK model in mice.

Parameter	Value	%CV	Unit	Description	Reference
L	0.12		mL/h	Total lymph flow	[12]
ISF	4.35		mL	Total interstitial flow volume	[12]
K_p_	0.156		-	Tissue-to-plasma concentration ratio	Experimental data
V_p_	1.74	6.5	mL	Plasma volume	Fitted data
V_lymph_	1.7		mL	Lymphatic volume	[12]
V_tumor_	0.5		mL	Tumor volume	Experimental data
CL_p_	0.0181	3.0	mL/h	Clearance	Fitted data
k_pt_	0.00269	0.85	1/h	Rate constant	Fitted data
k_tp_	0.0602	2.6	1/h	Rate constant	Fitted data
S_1_	0.950		-	Vascular reflection coefficients for V_tight_	[12]
S_2_	0.475	9.9	-	Vascular reflection coefficients for V_leaky_	Fitted data
S_L_	0.2		-	Lymphatic capillary reflection coefficients	[25]

**Table 4:** The optimized mass transition and condition for multiple reaction monitoring (MRM) analysis for therapeutic fusion proteins (TFP).

Protein	Peptide	Q1 (*m*/*z*)	Q3 (*m*/*z*)	Fragment	DP (V)	EP (V)	CE (eV)	CXP (V)
Human IgG4	SLSLSLGK	403.0 (charge: 2)	604.4	y6	121	10	17	54
	SLSLSLG[K]	407.0 (charge: 2)	612.4	y6	121	10	17	54
Interferon alpha	EDSILAVR	451.8 (charge: 2)	458.2	y4	76	10	23	44
	EDSILAV[R]	456.8 (charge: 2)	468.2	y4	76	10	23	44

Optimized mass transition and condition for MRM analysis for TFP. DP, EP, CE and CXP represents declustering potential, collision energy, and collision cell exit potential. [K] and [R] indicate ^13^C_6_^15^N_4_-R and ^13^C_6_^15^N_2_-K, respectively.
